# Effects of Anthropogenic Disturbance on Mammal Community Diversity and Activity Patterns: Evidence from the Jinfoshan and Jinyunshan National Nature Reserves, China

**DOI:** 10.3390/ani16050695

**Published:** 2026-02-24

**Authors:** Zeguang Guo, Hanyu Zhu, Jie He, Ling Shen, Wancai Xia

**Affiliations:** 1 Key Laboratory of Southwest China Wildlife Resources Conservation (Ministry of Education), China West Normal University, Nanchong 637009, China; 2 Key Laboratory of Conservation Biology of *Rhinopithecus roxellana*, China West Normal University, Nanchong 637009, China; 3 Yunnan Baima Snow Mountain National Nature Reserve Administration Bureau, Diqing 674499, China; 4 Chongqing Academy of Forestry, Chongqing 400036, China

**Keywords:** anthropogenic disturbance, camera trapping, mammal community, taxonomic diversity, functional diversity, diel activity pattern

## Abstract

Anthropogenic disturbance can reshape mammal communities through both long-term habitat modification and short-term direct human presence, yet these disturbance dimensions are often conflated. Using three consecutive years (2017–2019) of camera-trap data from two subtropical forest reserves in Chongqing, China (Jinfoshan and Jinyunshan), we evaluated the differential effects of human modification (HM) and human activity (HA) on mammal taxonomic diversity, functional diversity (MNTD and SES.MNTD), and diel activity patterns. HM and HA were not significantly correlated, indicating that they represent largely independent disturbance components in this system. Regression analyses showed that HM was significantly associated with reduced Shannon–Wiener diversity, while HA primarily influenced community evenness with a non-linear response (initial increase followed by decline at higher disturbance). In contrast, functional diversity metrics (MNTD and SES.MNTD) did not exhibit significant relationships with either HM or HA across the observed gradients, suggesting relative stability of trait dispersion at the community level. Activity analyses revealed guild-specific behavioral strategies: herbivores retained a predominantly crepuscular pattern but reduced activity during periods of high human presence, whereas omnivores displayed stronger temporal niche partitioning, becoming more nocturnal under higher disturbance intensity, particularly in Jinyunshan. Together, these results demonstrate that distinct disturbance types can affect mammal communities through different pathways and that integrating multidimensional diversity metrics with behavioral analyses can improve conservation planning in human-dominated landscapes.

## Simple Summary

Human disturbance is increasingly shaping wildlife communities, even inside protected areas. Using three years (2017–2019) of camera-trap monitoring in two subtropical forest reserves in Chongqing, China (Jinfoshan and Jinyunshan), we assessed how short-term direct human activity (HA) and long-term landscape human modification (HM) influence mammal diversity and daily activity patterns. Although the two reserves differ in elevation range and mammal diversity, HA and HM were largely independent across camera stations, allowing us to evaluate their distinct effects. We found that HM was associated with reduced mammal taxonomic diversity (Shannon–Wiener index), whereas HA mainly affected community evenness, showing a non-linear response (evenness increased at low disturbance but declined at high disturbance). Mammals also altered their behavior to reduce encounters with humans. Our results highlight that different disturbance dimensions affect mammal communities in different ways, and effective conservation should manage both habitat modification and direct human use within and around protected areas.

## 1. Introduction

Biodiversity loss is a major global crisis largely driven by human activities. It is estimated that around one million species are currently under threat of extinction, with many populations having declined sharply in recent decades. [1,2,3]. In the Anthropocene, human impacts are nearly ubiquitous: ~95% of terrestrial land has been modified, and most mountain vegetation loss has been linked to human activities, including substantial losses even within protected areas [4,5,6]. Rapid industrialization, urbanization, and agricultural expansion have accelerated habitat loss and declines of large-bodied wildlife, and mammals are often among the most sensitive groups to these pressures [2,7,8,9]. As a result, understanding how different forms of human pressure restructure mammal communities has become a central challenge in conservation science [10,11].

Human pressure is not a single process, and ecological responses may differ depending on whether disturbance reflects (i) long-term landscape modification that accumulates through land conversion and infrastructure, or (ii) short-term direct human presence that triggers immediate avoidance [11]. Yet many studies quantify disturbance using only one dimension (or merge multiple dimensions), making it difficult to disentangle mechanisms and to reconcile apparently inconsistent findings across systems [11]. Moreover, protected areas often contain internal gradients of human use and access, providing an opportunity to evaluate how different disturbance types jointly shape mammal communities while holding broad regional context relatively constant [3,12].

To capture these mechanisms, it is necessary to consider both community structure and behavioral adjustment. Biodiversity influences ecosystem functioning and services through complex pathways [12], but richness or abundance alone may fail to detect functionally meaningful changes in communities [12,13,14,15,16]. Functional diversity metrics, including mean nearest taxon distance (MNTD) and its standardized effect size (SES.MNTD), are widely used to quantify trait dispersion and infer assembly processes [17,18]. Mammal communities exposed to increasing human modification often show reduced MNTD and negative SES.MNTD values, consistent with functional homogenization driven by anthropogenic filtering [11]. At the same time, animals can respond rapidly to disturbance by altering diel activity schedules to reduce encounters with humans (temporal niche partitioning), often shifting activity away from periods of high human presence and, in many cases, toward nocturnality [11,19,20,21,22,23,24]. In addition, differences in biodiversity may also mediate how human disturbance regulates animal activity patterns. Camera trapping combined with kernel density estimation therefore provides a standard non-invasive framework for evaluating diel activity patterns and temporal overlap in medium-to-large mammals [25,26,27,28,29,30].

In this study, we focus on two subtropical forest reserves in Chongqing, China (Jinfoshan and Jinyunshan), where mammal communities are increasingly threatened by human activities despite formal protection [31,32,33,34,35]. During our survey, we found that the two reserves differed in biodiversity. Given that biodiversity differences may influence animals’ activity responses to human disturbance, we aimed to obtain robust conclusions by conducting parallel investigations across these two reserves. Importantly, large carnivores are absent in these systems, which helps reduce the confounding effects of predator-induced fear on activity schedules that can otherwise complicate attribution of activity shifts to human disturbance [36,37,38,39,40]. Using three years (2017–2019) of camera-trap data, we explicitly distinguish between long-term human modification of the landscape (HM) and short-term direct human activity (HA), and evaluate their effects on mammal taxonomic diversity and functional diversity (MNTD and SES.MNTD), as well as diel activity patterns [11]. We further test whether carnivores, herbivores, and omnivores exhibit contrasting avoidance strategies, with herbivores primarily adjusting activity timing to avoid periods of high human presence and omnivores showing stronger shifts toward nocturnality. Specifically, we test three hypotheses: (i) anthropogenic disturbance reduces mammal diversity across multiple dimensions (H1) [2,9,23]; (ii) HM and HA exert distinct effects on community diversity (H2) [5,34,35]; and (iii) carnivores, herbivores, and omnivores adopt divergent avoidance strategies (H3) [23,24]. By integrating multidimensional diversity metrics with activity rhythm analyses, this study aims to clarify how different disturbance types reshape mammal communities and to inform conservation in human-dominated landscapes [10,11].

## 2. Materials and Methods

**Figure 1:**
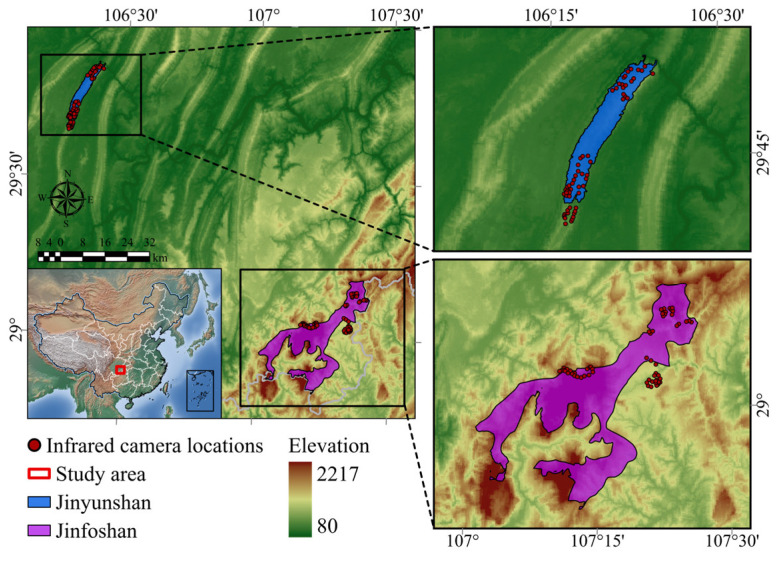
Study area and camera-trap survey design.

This study was conducted in two national nature reserves in Chongqing, China: Jinfoshan National Nature Reserve (hereafter Jinfoshan) and Jinyunshan National Nature Reserve (hereafter Jinyunshan) (Figure 1). Jinfoshan is located in Nanchuan District (28°50′–29°20′ N, 107°00′–107°20′ E) and covers approximately 418.5 km^2^, representing the largest protected area within the Chongqing metropolitan region. Jinyunshan is situated across Beibei District, Shapingba District, and Bishan County (29°48′–29°52′ N, 106°20′–106°24′ E) with an area of 76 km^2^. Both reserves are characterized by a subtropical humid monsoon climate. The two reserves are approximately 105.26 km apart. Elevation ranges from 714 to 1846 m in Jinfoshan and from 265 to 856 m in Jinyunshan. During our survey, we found that the two reserves differed in mammal biodiversity, with 13 species recorded in Jinfoshan and 9 species recorded in Jinyunshan.

### 2.1. Camera-Trap Survey Design and Data Processing

We conducted a three-year camera-trap survey from 2017 to 2019 in Jinfoshan and Jinyunshan. To capture variation in mammal communities along gradients of anthropogenic disturbance rather than to compile a complete species inventory, camera-trap locations were strategically selected based on elevation, vegetation type, accessibility, reserve zoning, and proximity to human activities. In each reserve and its surrounding areas, we established three sampling blocks spanning gradients of human activity intensity and distance to villages, enabling explicit comparisons between sites with differing disturbance levels inside and outside protected areas. Within each block, 20 camera-trap stations were deployed, resulting in a total of 120 camera traps across the two reserves (Figure 1).

In this study, we used Ltl Acorn infrared camera traps (Ltl Acorn, Shenzhen Ltl Acorn Electronics Co., Ltd., Shenzhen, China). Camera placement was designed to maximize detection probability by targeting locations likely to be used by mammals, such as areas near water sources, sites with abundant food resources, and trails or places showing signs of animal activity. Camera stations were spaced at least 500 m apart. Given the absence of large-bodied mammals in both reserves, cameras were mounted at approximately 80 cm above ground to improve image quality [40]. All cameras were configured in a combined photo–video mode, capturing three consecutive photographs followed by a 15 s video clip per trigger event [41]. The trigger interval was set to 1 min, sensitivity was set to “medium”, and cameras operated continuously for 24 h per day.

Cameras were checked approximately every three months to replace batteries and memory cards, and to ensure normal operation. Retrieved images were archived according to collection date, and each camera station was assigned a unique identifier. Raw data were backed up prior to processing. Continuous blank images were removed, and metadata (e.g., timestamp and camera ID) were extracted using the “Biophoto” program. Independent detection events were defined as records of the same species at the same camera station separated by at least 30 min [42,43].

### 2.2. Quantifying Anthropogenic Disturbance

Because mammals are subjected to multiple forms of human pressure [44], we classified anthropogenic disturbance into two components: short-term human activity (HA) and long-term human modification (HM). Human activity was quantified using camera-trap records of humans, poultry, and livestock, which were treated as HA events. HA intensity was measured using the species abundance index (SAI; relative abundance index) [45]. In contrast, long-term human modification (HM) was obtained from the Global Human Modification (gHM) dataset (doi:10.1111/gcb.14549), which integrates the spatial extent and influence of 13 anthropogenic stressors (e.g., human settlements, agriculture, and transportation) with a median year of 2016. The dataset provides a cumulative measure of terrestrial modification at a 1 km^2^ resolution, ranging from 0.0 (no modification) to 1.0 (complete modification). HM values for each camera station were extracted using ArcGIS 10.8 (ESRI, Redlands, CA, USA, 2020).

We then calculated the Pearson correlation coefficient between HA and HM to evaluate their spatial dependency. Differences in anthropogenic disturbance between the two reserves were assessed using *t*-tests, with statistical significance set at *p* < 0.05.

### 2.3. Taxonomic and Functional Diversity

To characterize taxonomic diversity, we calculated the Shannon–Wiener diversity index and Pielou’s evenness index, representing community diversity and evenness, respectively [46]. To quantify functional diversity, we selected three commonly used traits reflecting mammal responses to environmental conditions and their ecological roles: adult body mass, trophic level, and activity cycle [47,48]. Based on the PanTHERIA database, trophic level was classified into three categories: (1) herbivore, (2) omnivore, and (3) carnivore. Activity cycle was grouped into three categories: (1) nocturnal only, (2) nocturnal/crepuscular–cathemeral–crepuscular or diurnal/crepuscular, and (3) diurnal only. Trait data were compiled from published trait datasets [49]. Because these traits included both continuous and categorical variables, we calculated pairwise functional distances using Gower distance to construct a functional trait distance matrix [50]. Based on this matrix, we calculated the mean nearest taxon distance (MNTD) for each camera station using the “picante” package (version 1.8.2). To control for the influence of species richness, we further estimated the standardized effect size of MNTD (SES.MNTD) using a null model with 999 randomizations. MNTD represents the mean distance between each species and its most functionally similar neighbor, reflecting within-community functional similarity, whereas SES.MNTD compares observed values against null expectations to identify non-random trait structuring. In this study, higher MNTD and SES.MNTD values indicate increased functional overdispersion, suggesting niche differentiation and competitive exclusion; lower values indicate functional clustering, which may reflect potential trait filtering driven by environmental constraints or anthropogenic disturbance [11]. We used regression models to evaluate the effects of HM and HA on taxonomic and functional diversity metrics separately for Jinfoshan and Jinyunshan. All taxonomic diversity indices were calculated using the “vegan” package in R (version 2.6-4).

### 2.4. Activity Pattern Analysis

We examined diel activity patterns of herbivorous and omnivorous mammals in response to anthropogenic disturbance using the R package “overlap” (version 0.3.3) [27]. Camera-trap timestamps were first converted to solar time and then transformed into radian values (0–2π) to meet the assumptions of circular statistical analysis. Nonparametric kernel density estimation was used to construct probability density functions describing diel activity patterns for disturbance events and mammal detections.

To quantify temporal overlap between anthropogenic disturbance and mammal activity, we calculated the coefficient of overlap (Δ), which ranges from 0 (no overlap) to 1 (complete overlap). The estimator was selected according to sample size, and 95% confidence intervals were derived from 1000 bootstrap replicates. Changes in overlap provide insights into behavioral responses: reduced overlap indicates temporal avoidance of anthropogenic disturbance through activity shifts, whereas increased overlap suggests limited temporal adjustment or possible habituation/adaptive responses [27].

## 3. Results

### 3.1. Mammal Surveys

During the three-year camera-trapping survey (2017–2019), the 60 camera traps deployed in Jinfoshan accumulated a total of 24,603 camera-trap days, yielding 2976 independent mammal records and 548 independent records of human activity. In Jinyunshan, 60 camera traps accumulated 19,738 camera-trap days, producing 703 independent mammal records and 418 independent records of human activity.

In total, we recorded 15 mammal species belonging to 4 orders and 10 families across the two reserves. These species included six herbivores, namely *Lepus tolai*, *Macaca mulatta*, *Trachypithecus francoisi*, *Muntiacus reevesi*, *Moschus berezovskii*, and *Elaphodus cephalophus*; five omnivores, including *Meles leucurus*, *Arctonyx collaris*, *Atherurus macrourus*, *Sus scrofa*, and *Paguma larvata*; and four small carnivores, including *Prionodon pardicolor*, *Martes flavigula*, *Prionailurus bengalensis*, and *Mustela sibirica* (Table 1).

### 3.2. Diversity and Anthropogenic Disturbance Difference

**Figure 2:**
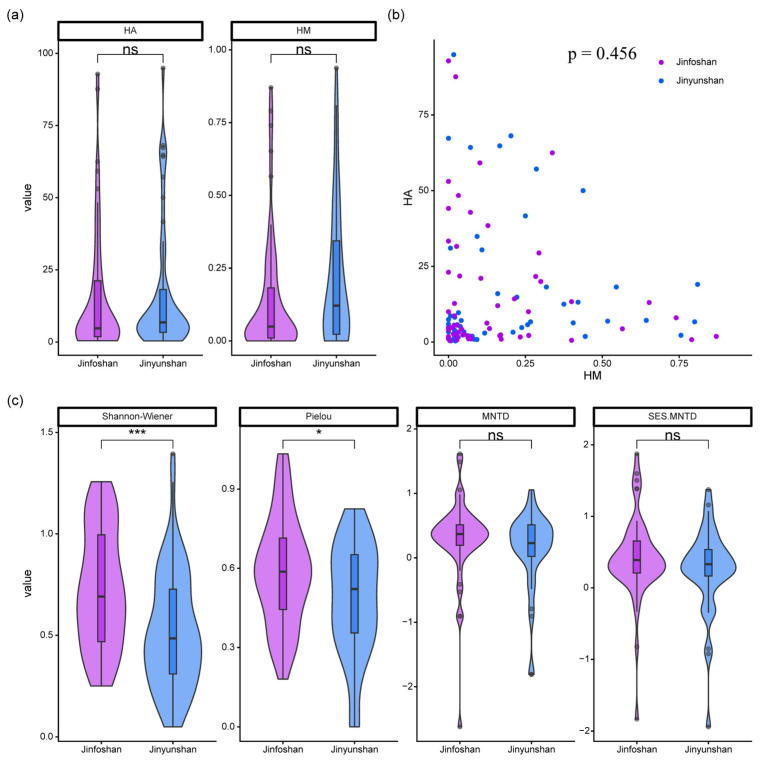
Differences in mammal diversity and anthropogenic disturbance between Jinfoshan and Jinyunshan National Nature Reserves. (**a**) Comparisons of human activity (HA) and human modification (HM) between the two reserves. (**b**) Relationships between HA and HM across camera-trap stations. (**c**) Comparisons of Shannon–Wiener diversity, Pielou’s evenness, MNTD, and SES.MNTD between the two reserves. Significance levels are indicated as follows: *** *p* < 0.001; * *p* < 0.05; ns, not significant.

Taxonomic diversity differed significantly between the two reserves. Both the Shannon–Wiener diversity index and Pielou’s evenness were significantly higher in Jinfoshan than in Jinyunshan, whereas functional diversity did not differ significantly between reserves (Figure 2c).

Neither human modification (HM) nor human activity (HA) showed significant differences between Jinfoshan and Jinyunshan (Figure 2a). In addition, HM and HA were not significantly correlated within either reserve (Figure 2b), indicating that long-term landscape modification and short-term direct human presence represented largely independent disturbance dimensions in the study system.

### 3.3. Effects of Anthropogenic Disturbance on Mammal Biodiversity

**Figure 3:**
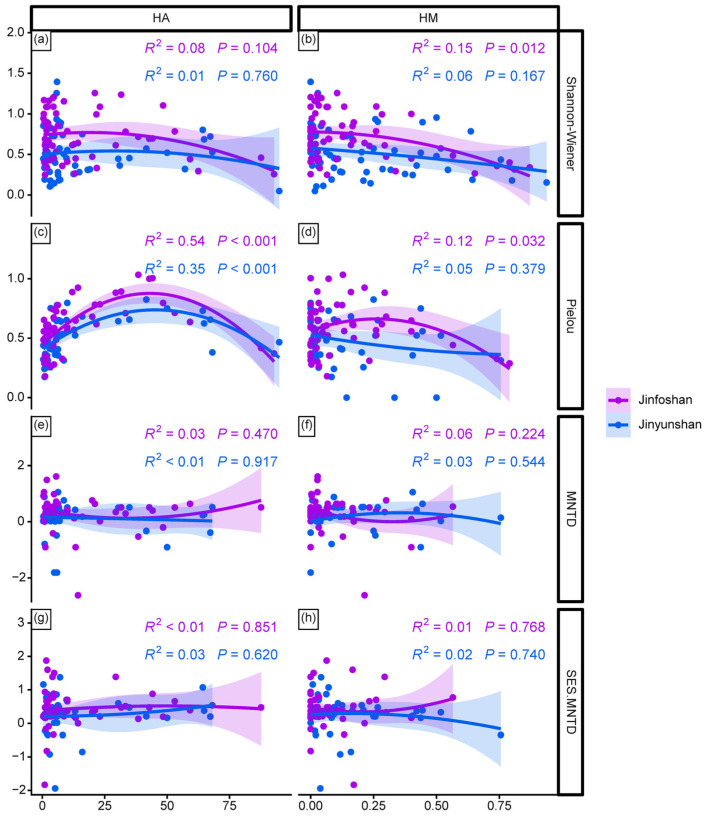
Effects of human activity (HA) and human modification (HM) on mammal taxonomic diversity (**a**–**d**) and functional diversity (**e**–**h**) across camera-trap stations in the two reserves.

At the taxonomic diversity level, in Jinfoshan, Pielou’s evenness responded non-linearly to both HA and HM, increasing at low disturbance levels and decreasing as disturbance intensified (Figure 3c,d). HM was also significantly negatively associated with the Shannon–Wiener diversity index (Figure 3b). In Jinyunshan, HA was the only significant predictor of Pielou’s evenness, again showing a non-linear (hump-shaped) relationship (Figure 3c).

For functional diversity metrics, neither HA nor HM showed significant effects on MNTD or SES.MNTD (Figure 3e–h), indicating that functional diversity remained relatively stable across the observed disturbance gradients.

### 3.4. Effects of Human Disturbance on Diel Activity Patterns of Herbivorous and Omnivorous Mammals

**Figure 4:**
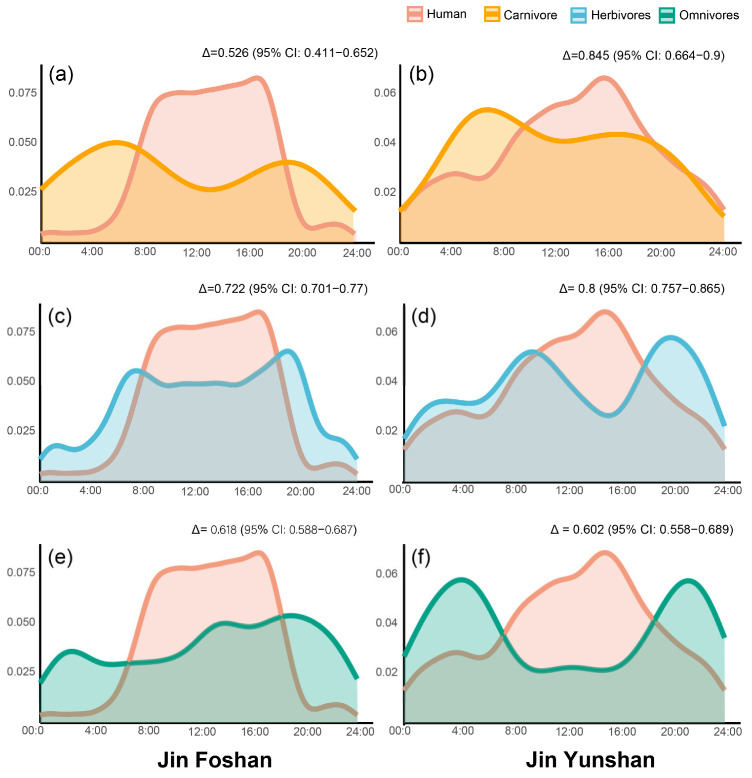
Temporal overlap between human activity and mammal diel activity patterns in Jinfoshan and Jinyunshan National Nature Reserves. Panels show the overlap between diel activity patterns of human activity and (**a**,**b**) carnivores, (**c**,**d**) herbivores, and (**e**,**f**) omnivores.

Human diel activity in both reserves peaked at approximately 16:00; in comparison, nocturnal human activity was lower in Jinfoshan. Across both reserves, carnivorous, herbivorous, and omnivorous mammals generally exhibited avoidance of human activity. Small carnivorous mammals tended to peak in activity at around 07:00. The avoidance effect was stronger in Jinfoshan (Δ = 0.526, 95% CI: 0.411–0.652; Figure 4a), whereas overlap with human activity was higher in Jinyunshan (Δ = 0.845, 95% CI: 0.664–0.9; Figure 4b).

Herbivorous mammals exhibited a typical crepuscular activity pattern, with two primary peaks occurring in the early morning (around 08:00) and evening (around 20:00). Their temporal overlap with human activity was relatively high (Δ = 0.722 in Jinyunshan and Δ = 0.800 in Jinfoshan; Figure 4b,c); nevertheless, herbivore activity declined during periods of elevated human presence.

Omnivorous mammals displayed more divergent patterns between the two reserves. In Jinfoshan, omnivore activity was multimodal, with prominent peaks in the afternoon (13:00–14:00) and at night (19:00–20:00), and a smaller peak during 02:00–03:00. The overlap coefficient between omnivores and human activity was Δ = 0.618 (95% CI: 0.588–0.687; Figure 4e). In contrast, omnivorous mammals in Jinyunshan showed a strongly nocturnal activity pattern, with activity peaks concentrated in the early morning (04:00–05:00) and late night (21:00–22:00). This pattern suggested a clear temporal partitioning from human activity, with an overlap coefficient of Δ = 0.602 (95% CI: 0.558–0.689; Figure 4d).

## 4. Discussion

As expected, our results indicate that different components of anthropogenic disturbance can exert distinct effects on mammal community biodiversity. Overall, our findings provide partial to strong support for the three hypotheses (H1–H3) proposed in this study, underscoring the multidimensional and guild-specific nature of mammal responses to human disturbance. Short-term direct human activity (HA) was more strongly associated with changes in community evenness and diel activity (consistent with behavioral avoidance), whereas long-term human modification (HM) showed a clearer relationship with taxonomic diversity. Together, these patterns suggest that direct human presence and accumulated landscape modification may influence mammal communities through different—yet complementary—pathways.

### 4.1. Hypothesis 1: Effects of Anthropogenic Disturbance on Multidimensional Biodiversity

HA exhibited non-linear effects on both species diversity and evenness: low levels of human disturbance were associated with higher evenness, whereas evenness declined rapidly at higher levels of HA. This pattern may reflect that short-term human presence disproportionately affects particular taxa, especially high-abundance (dominant) species, thereby reshaping relative abundances and community evenness. In contrast, long-term disturbance may exert a stronger influence on overall taxonomic diversity. The significant negative relationship between HM and the Shannon–Wiener index corroborates global evidence that habitat fragmentation and anthropogenic landscape transformation are major drivers of biodiversity loss [5,16,36]. These results partially support our H1 that anthropogenic disturbance reduces mammal diversity. More importantly, HM appeared to impose stronger constraints on taxonomic diversity than on functional diversity, which deviates from our expectation. Previous studies have shown that persistent anthropogenic modification acts as an environmental filter that selectively excludes species with certain functional traits, ultimately favoring communities composed of functionally similar species [21,50]. For example, habitat fragmentation disproportionately eliminates large-bodied species that require extensive contiguous habitats [13,51,52,53], while road construction exerts stronger impacts on disturbance-sensitive species [54,55]. However, our results did not support a clear trait-filtering signal. One plausible explanation is that human disturbance in our system may impose a broadly negative pressure across mammals, affecting species in a more general manner rather than consistently excluding specific trait syndromes. Together, these patterns suggest that anthropogenic disturbance may have stronger impacts on numerically dominant species (thereby altering abundance structure and evenness) than on particular functional traits.

### 4.2. Hypothesis 2: Contrasting Effects of Human Modification and Human Activity

Regression analyses showed that HA had a stronger effect on community evenness, whereas long-term HM was associated with declines in species diversity; moreover, this effect may be more pronounced in areas with higher baseline diversity. This clear divergence provides strong support for our H2 that different disturbance types exert distinct impacts on mammal communities. As a short-term and intermittent disturbance, HA primarily affects mammals through immediate behavioral interference, altering local occurrence and activity patterns [23]. Such effects may act first on numerically dominant species, thereby reducing evenness by disproportionately suppressing the most abundant taxa. In contrast, long-term HM may lead to the directional exclusion of species that are more sensitive to human disturbance, thereby constraining their distributions and reducing overall diversity [5,51,52,56]. Spatially, HM and HA were only weakly correlated, indicating that areas with relatively low landscape modification may still experience substantial ecological pressure due to frequent direct human presence [44,57]. This decoupling highlights the need to explicitly distinguish between structural habitat modification and direct human disturbance when assessing anthropogenic impacts and designing conservation strategies [58,59,60].

### 4.3. Hypothesis 3: Guild-Specific Behavioral Responses to Disturbance

At the behavioral level, herbivorous mammals exhibited contrasting adaptive strategies across disturbance gradients. In Jinfoshan, herbivores maintained a typical crepuscular activity pattern and reduced activity during peak human presence, rather than abandoning habitats or fundamentally shifting circadian rhythms, reflecting a conservative behavioral strategy under moderate disturbance [23]. In contrast, herbivores in Jinyunshan displayed more complex behavioral adjustments. Although temporal overlap with human activity was higher than in Jinfoshan, herbivores adopted a multimodal activity pattern, enabling continued resource use under intense disturbance [27]. This seemingly paradoxical pattern likely reflects habitat constraints: Jinyunshan may have reduced and fragmented suitable habitats, forcing herbivores to exploit areas overlapping with human activity to access essential resources [41,42]. Fine-scale temporal adjustments [23] and increased vigilance behaviors [61] may help mitigate direct conflict risks under such conditions.

Herbivorous mammals appeared more tolerant of human modification than omnivores, a pattern closely linked to their ecological characteristics. Previous studies have shown that generalist herbivores, such as the muntjac (*Muntiacus reevesi*), one of the most common species in both reserves, possess broad dietary niches and flexible habitat use that facilitate persistence in moderately modified landscapes [38,61,62]. Additionally, the “human shield” hypothesis suggests that some herbivores may actively select areas near human activity to reduce predation risk [62]. These findings support our H3 by demonstrating that herbivores primarily rely on fine-scale temporal adjustments rather than wholesale shifts in activity schedules.

In contrast, omnivorous mammals exhibited more pronounced divergence in activity patterns between the two reserves. In Jinfoshan, omnivores maintained a multimodal activity pattern with moderate temporal overlap with humans, suggesting that under relatively low disturbance, omnivores can coexist with human activity through partial temporal adjustments while largely retaining natural activity rhythms. In contrast, omnivores in Jinyunshan displayed a strictly nocturnal pattern under high anthropogenic pressure, concentrating activity during periods of minimal human presence and achieving strong temporal niche partitioning. This shift toward nocturnality has been widely documented as a common response of wildlife to increasing human activity [23], and our findings reinforce this global pattern. Temporal niche partitioning allows omnivores to reduce direct temporal competition with humans [55], facilitating coexistence in shared landscapes. However, forced nocturnality may restrict access to diurnally abundant food resources, potentially leading to simplified diets and compromised health [63,64]. During resource-scarce seasons, such temporal constraints may increase energetic stress and ultimately influence population dynamics [65]. Therefore, long-term conservation of omnivores requires integrated management strategies that combine habitat protection with regulation of human activities, ensuring the availability of sufficient diurnal resources and activity space [66].

### 4.4. Limitations and Future Directions

In this study, we used the gHM dataset at a 1 km spatial resolution to quantify long-term human modification, which does not match the spatial grain of the short-term human activity metric (HA) derived from camera-trap observations. This mismatch may introduce bias by smoothing fine-scale variation in disturbance and by creating spatial misalignment between site-level camera records and pixel-level HM values. Moreover, HA and HM were not fully aligned temporally: our camera-trap data were collected during 2017–2019, whereas the HM layer represents conditions around 2016. This temporal offset may add uncertainty to our estimates. Nevertheless, because HM is intended as a proxy for cumulative, long-term human influence, the resulting uncertainty is likely to be relatively constrained compared with short-term fluctuations in direct human presence.

Although large carnivores were absent in our system, interactions with small carnivores and interspecific dynamics may still affect observed activity patterns of herbivores and omnivores. As highlighted by Veech et al. (2006), species interactions can significantly mediate wildlife responses to anthropogenic disturbance [66]. In addition, avoidance behavior in heavily disturbed areas may reduce detection probability, potentially biasing estimates of species occurrence and activity. Future studies integrating occupancy modeling, longer monitoring periods, and complementary methods such as telemetry would help refine inference and further disentangle behavioral and demographic processes.

## 5. Conclusions

Our results demonstrate that HA exerted non-linear effects on species diversity and evenness: community evenness increased under low levels of human activity but declined rapidly as HA intensified, whereas long-term HM was associated with decreases in species diversity, an effect that was more pronounced in areas with higher baseline species richness. Furthermore, mammals with different feeding habits exhibited distinct adaptive strategies in response to anthropogenic pressure. Herbivores tended to minimize direct conflict with humans through fine-scale temporal adjustments, whereas omnivores primarily adopted more nocturnal activity to avoid human presence. These findings underscore the importance of distinguishing among disturbance types and recognizing feeding-habit-specific responses when designing conservation and management strategies. Integrative approaches that simultaneously address habitat modification and direct human activity are essential for mitigating the negative impacts of human disturbance on biodiversity.

## Figures and Tables

**Table 1:** Species list and dietary guilds for Jinfoshan and Jinyunshan.

Species	Dietary	Jinfoshan	Jinyunshan
* Prionodon pardicolor *	omnivore	✓	
* Prionailurus bengalensis *	carnivore	✓	✓
*Meles leucurus*	omnivore		✓
* Trachypithecus francoisi *	herbivore	✓	
* Paguma larvata *	omnivore	✓	✓
*Martes flavigula*	carnivore	✓	
* Mustela sibirica *	carnivore	✓	✓
* Moschus berezovskii *	herbivore	✓	
* Elaphodus cephalophus *	herbivore	✓	✓
* Lepus tolai *	herbivore		✓
* Macaca mulatta *	herbivore	✓	
* Muntiacus reevesi *	herbivore	✓	✓
* Sus scrofa *	omnivore	✓	✓
* Atherurus macrourus *	omnivore	✓	
* Arctonyx collaris *	omnivore	✓	✓

## Data Availability

The data presented in this study are not public but are available on request from the corresponding author due to them being part of an ongoing research project.

## References

[1] Bongaarts J. (2019). Summary for policymakers of the global assessment report on biodiversity and ecosystem services of the Intergovernmental Science policy Platform on Biodiversity and Ecosystem Services. Popul. Dev. Rev..

[2] Keck F., Peller T., Alther R., Barouillet C., Blackman R., Capo E., Chonova T., Couton M., Fehlinger L., Kirschner D. (2025). The global human impact on biodiversity. Nature.

[3] Newbold T., Hudson L.N., Hill S.L.L., Contu S., Lysenko I., Senior R.A., Börger L., Bennett D.J., Choimes A., Collen B. (2015). Global effects of land use on local terrestrial biodiversity. Nature.

[4] Lewis S.L., Maslin M.A. (2015). Defining the Anthropocene. Nature.

[5] Kennedy C.M., Oakleaf J.R., Theobald D.M., Baruch-Mordo S., Kiesecker J. (2019). Managing the middle: A shift in conservation priorities based on the global human modification gradient. Glob. Change Biol..

[6] Laurance W.F., Carolina Useche D., Rendeiro J., Kalka M., Bradshaw C.J.A., Sloan S.P., Laurance S.G., Campbell M., Abernethy K., Alvarez P. (2012). Averting biodiversity collapse in tropical forest protected areas. Nature.

[7] Su G., Logez M., Xu J., Tao S., Villéger S., Brosse S. (2021). Human impacts on global freshwater fish biodiversity. Science.

[8] Wittische J., Heckbert S., James P.M.A., Burton A.C., Fisher J.T. (2021). Community-level modelling of boreal forest mammal distribution in an oil sands landscape. Sci. Total Environ..

[9] Doherty T.S., Hays G.C., Driscoll D.A. (2021). Human disturbance causes widespread disruption of animal movement. Nat. Ecol. Evol..

[10] Dirzo R., Young H.S., Galetti M., Ceballos G., Isaac N.J.B., Collen B. (2014). Defaunation in the Anthropocene. Science.

[11] Li X., Hu W., Bleisch W.V., Li Q., Wang H., Lu W., Sun J., Zhang F., Ti B., Jiang X. (2022). Functional diversity loss and change in nocturnal behavior of mammals under anthropogenic disturbance. Conserv. Biol..

[12] Hooper D.U., Chapin F., Ewel J.J., Hector A., Inchausti P., Lavorel S., Lawton J.H., Lodge D.M., Loreau M., Naeem S. (2005). Effects of biodiversity on ecosystem functioning: A consensus of current knowledge. Ecol. Monogr..

[13] Gibson L., Lee T.M., Koh L.P., Brook B.W., Gardner T.A., Barlow J., Peres C.A., Bradshaw C.J.A., Laurance W.F., Lovejoy T.E. (2011). Primary forests are irreplaceable for sustaining tropical biodiversity. Nature.

[14] Petchey O.L., Gaston K.J. (2006). Functional diversity: Back to basics and looking forward. Ecol. Lett..

[15] Laméris D.W., Tagg N., Kuenbou J.K., Sterck E.H.M., Willie J. (2020). Drivers affecting mammal community structure and functional diversity under varied conservation efforts in a tropical rainforest in Cameroon. Anim. Conserv..

[16] Weideman E.A., Slingsby J.A., Thomson R.L., Coetzee B.T.W. (2020). Land cover change homogenizes functional and phylogenetic diversity within and among African savanna bird assemblages. Landsc. Ecol..

[17] Díaz S., Fargione J., Chapin F.S., Tilman D. (2006). Biodiversity Loss Threatens Human Well-Being. PLoS Biol..

[18] Revell L.J. (2010). Phylogenetic signal and linear regression on species data. Methods Ecol. Evol..

[19] Kembel S.W., Cowan P.D., Helmus M.R., Cornwell W.K., Morlon H., Ackerly D.D., Blomberg S.P., Webb C.O. (2010). Picante: R tools for integrating phylogenies and ecology. Bioinformatics.

[20] Zhan C., Chen X., Li B., Chen C., Wang Y. (2025). Human-Driven Extinctions Rapidly Alter the Island Biodiversity Patterns of Large and Medium-Sized Mammals in the Largest Archipelago of China. Divers. Distrib..

[21] Mori A.S., Isbell F., Seidl R. (2018). β-Diversity, Community Assembly, and Ecosystem Functioning. Trends Ecol. Evol..

[22] Lirong Z., Ibrahim M.A., Yuanyuan L., Limin W., Shu F., Dongming L. (2023). Effects of constant light and dark conditions on the locomotor activity, body mass, and body temperature rhythms of Eurasian Tree Sparrows (*Passer montanus*). Avian Res..

[23] Gaynor K.M., Hojnowski C.E., Carter N.H., Brashares J.S. (2018). The influence of human disturbance on wildlife nocturnality. Science.

[24] Takashi I., Daishi H., Tomoya S. (2022). Impact of human disturbance in Japan on the distribution and diel activity pattern of terrestrial mammals. J. Nat. Conserv..

[25] O’Connell A.F., Nichols J.D., Karanth K.U. (2010). Camera Traps in Animal Ecology: Methods and Analyses.

[26] Rowcliffe J.M., Carbone C. (2008). Surveys using camera traps: Are we looking to a brighter future?. Anim. Conserv..

[27] Ridout M.S., Linkie M. (2009). Estimating overlap of daily activity patterns from camera trap data. J. Agric. Biol. Environ. Stat..

[28] Silverman B.W. (1986). Density estimation for statistics and data analysis. Monographs on Statistics and Applied Probability.

[29] Rowcliffe J.M., Kays R., Kranstauber B., Carbone C., Jansen P.A. (2014). Quantifying levels of animal activity using camera trap data. Methods Ecol. Evol..

[30] Ripple W.J., Estes J.A., Beschta R.L., Wilmers C.C., Ritchie E.G., Hebblewhite M., Berger J., Elmhagen B., Letnic M., Nelson M.P. (2014). Status and Ecological Effects of the World’s Largest Carnivores. Science.

[31] Estes J.A., Terborgh J., Brashares J.S., Power M.E., Berger J., Bond W.J., Carpenter S.R., Essington T.E., Holt R.D., Jackson J.B.C. (2011). Trophic Downgrading of Planet Earth. Science.

[32] Austin J.G., Scott C., Rory P.W., Steven J.C. (2017). Energy Landscapes and the Landscape of Fear. Trends Ecol. Evol..

[33] Gaynor K.M., Brown J.S., Middleton A.D., Power M.E., Brashares J.S. (2019). Landscapes of Fear: Spatial Patterns of Risk Perception and Response. Trends Ecol. Evol..

[34] Suraci J.P., Clinchy M., Zanette L.Y., Wilmers C.C. (2019). Fear of humans as apex predators has landscape-scale impacts from mountain lions to mice. Ecol. Lett..

[35] Clinchy M., Zanette L.Y., Roberts D., Suraci J.P., Buesching C.D., Newman C., Macdonald D.W. (2016). Fear of the human “super predator” far exceeds the fear of large carnivores in a model mesocarnivore. Behav. Ecol..

[36] Gray C.L., Hill S.L.L., Newbold T., Hudson L.N., Börger L., Contu S., Hoskins A.J., Ferrier S., Purvis A., Scharlemann J.P.W. (2016). Local biodiversity is higher inside than outside terrestrial protected areas worldwide. Nat. Commun..

[37] Ewart H.E., Pasqualotto N., Paolino R.M., Jensen K., Chiarello A.G. (2024). Effects of anthropogenic disturbance and land cover protection on the behavioural patterns and abundance of Brazilian mammals. Glob. Ecol. Conserv..

[38] Yang C., Xu H., Li Q., Wang X., Tang B., Chen J., Tu W., Zhang Y., Shi T., Chen M. (2025). Global loss of mountain vegetated landscapes and its impact on biodiversity conservation. Nat. Commun..

[39] Rija A.A., Critchlow R., Thomas C.D., Beale C.M. (2020). Global extent and drivers of mammal population declines in protected areas under illegal hunting pressure. PLoS ONE.

[40] Chen Y., Xiang Z., Wang X., Xiao W., Xiao Z., Ren B., He C., Sang C., Li H., Li M. (2015). Preliminary Study of the Newly Discovered Primate Species Rhinopithecus strykeri at Pianma, Yunnan, China Using Infrared Camera Traps. Int. J. Primatol..

[41] Zhang M., Tang C., Zhang Q., Zhan C., Chen C., Wang Y. (2022). Selective extinction and habitat nestedness are the main drivers of lizard nestedness in the Zhoushan Archipelago. Curr. Zool..

[42] Li Z., Tang Z., Xu Y., Wang Y., Duan Z., Liu X., Wang P., Yang J., Chen W., Prins H.H.T. (2021). Habitat Use and Activity Patterns of Mammals and Birds in Relation to Temperature and Vegetation Cover in the Alpine Ecosystem of Southwestern China with Camera-Trapping Monitoring. Animals.

[43] Fida T., Ahmad F., Bosso L., Ali N., Din S.U., Kabir M. (2024). Distribution, diel activity patterns and human-bear interactions of the Himalayan brown bear (*Ursus arctos isabellinus*) in the Deosai National Park, Pakistan. Mammal Res..

[44] Crain C.M., Kroeker K., Halpern B.S. (2008). Interactive and cumulative effects of multiple human stressors in marine systems. Ecol. Lett..

[45] Carbone C., Christie S., Conforti K., Coulson T., Franklin N., Ginsberg J.R., Griffiths M., Holden J., Kawanishi K., Kinnaird M. (2001). The use of photographic rates to estimate densities of tigers and other cryptic mammals. Anim. Conserv..

[46] Magurran A.E. (1988). Ecological Diversity and Its Measurement.

[47] Fountain-Jones N.M., Jordan G.J., Burridge C.P., Wardlaw T.J., Baker T.P. (2017). Trophic position determines functional and phylogenetic recovery after disturbance within a community. Funct. Ecol..

[48] Soria C.D., Pacifici M., Di Marco M., Stephen S.M., Rondinini C. (2021). COMBINE: A coalesced mammal database of intrinsic and extrinsic traits. Ecology.

[49] Zhu P., Liu W., Zhang X., Li M., Liu G., Yu Y., Li Z., Li X., Du J., Wang X. (2023). Correlated evolution of social organization and lifespan in mammals. Nat. Commun..

[50] Laliberté E., Legendre P. (2010). A distance-based framework for measuring functional diversity from multiple traits. Ecology.

[51] Fahrig L. (2003). Effects of Habitat Fragmentation on Biodiversity. Annu. Rev. Ecol. Evol. Syst..

[52] Wilman H., Belmaker J., Simpson J.E., Rosa C.D.L., Rivadeneira M.M., Jetz W. (2014). EltonTraits 1.0: Species-level foraging attributes of the world’s birds and mammals. Ecology.

[53] Crooks K.R., Burdett C.L., Theobald D.M., King S.R.B., Di Marco M., Rondinini C., Boitani L. (2017). Quantification of habitat fragmentation reveals extinction risk in terrestrial mammals. Proc. Natl. Acad. Sci. USA.

[54] Ana B.-L., Rob A., Pita A.V. (2010). The impacts of roads and other infrastructure on mammal and bird populations: A meta-analysis. Biol. Conserv..

[55] Carter N.H., Shrestha B.K., Karki J.B., Pradhan N.M., Liu J. (2012). Coexistence between wildlife and humans at fine spatial scales. Proc. Natl. Acad. Sci. USA.

[56] Chapin F.S., Zavaleta E.S., Eviner V.T., Naylor R.L., Vitousek P.M., Reynolds H.L., Hooper D.U., Lavorel S., Sala O.E., Hobbie S.E. (2000). Consequences of changing biodiversity. Nature.

[57] Thiel D., Jenni-Eiermann S., Braunisch V., Palme R., Jenni L. (2008). Ski Tourism Affects Habitat Use and Evokes a Physiological Stress Response in Capercaillie Tetrao urogallus: A New Methodological Approach. J. Appl. Ecol..

[58] Wang Y., Smith J.A., Wilmers C.C. (2017). Residential development alters behavior, movement, and energetics in an apex predator, the puma. PLoS ONE.

[59] Tucker M.A., Böhning-Gaese K., Fagan W.F., Fryxell J.M., Van Moorter B., Alberts S.C., Ali A.H., Allen A.M., Attias N., Avgar T. (2018). Moving in the Anthropocene: Global reductions in terrestrial mammalian movements. Science.

[60] Ciuti S., Northrup J.M., Muhly T.B., Simi S., Musiani M., Pitt J.A., Boyce M.S. (2012). Effects of Humans on Behaviour of Wildlife Exceed Those of Natural Predators in a Landscape of Fear. PLoS ONE.

[61] Li X., Bleisch W.V., Liu X., Hu W., Jiang X. (2020). Human disturbance and prey occupancy as predictors of carnivore richness and biomass in a Himalayan hotspot. Anim. Conserv..

[62] Berger J. (2007). Fear, human shields and the redistribution of prey and predators in protected areas. Biol. Lett..

[63] Cox D.T.C., Gardner A.S., Gaston K.J. (2021). Diel niche variation in mammals associated with expanded trait space. Nat. Commun..

[64] Middleton A.D., Kauffman M.J., McWhirter D.E., Cook J.G., Cook R.C., Nelson A.A., Jimenez M.D., Klaver R.W. (2013). Animal migration amid shifting patterns of phenology and predation: Lessons from a Yellowstone elk herd. Ecology.

[65] Gaynor K., Cherry M., Gilbert S., Kohl M., Larson C., Newsome T., Prugh L., Suraci J., Young J., Smith J. (2020). An applied ecology of fear framework: Linking theory to conservation practice. Anim. Conserv..

[66] Veech J.A. (2006). A probability-based analysis of temporal and spatial co-occurrence in grassland birds. J. Biogeogr..

